# Epidemiology, Staging, and Management of Multiple Myeloma

**DOI:** 10.3390/medsci9010003

**Published:** 2021-01-20

**Authors:** Sandeep Anand Padala, Adam Barsouk, Alexander Barsouk, Prashanth Rawla, Anusha Vakiti, Ravindra Kolhe, Vamsi Kota, Germame Hailegiorgis Ajebo

**Affiliations:** 1 Department of Medicine, Division of Nephrology, Hypertension and Transplant, Medical College of Georgia, Augusta University, Augusta, GA 30912, USA; 2 Sidney Kimmel Cancer Center, Jefferson University, Philadelphia, PA 19107, USA; adambarsouk@comcast.net; 3 Hematology-Oncology, Allegheny Health Network, Pittsburgh, PA 15212, USA; alexbarsouk@comcast.net; 4 Department of Internal Medicine, Sovah Health, Martinsville, VA 24112, USA; rawlap@gmail.com; 5 Department of Medicine, Hematology-Oncology, Medical College of Georgia, Augusta University, Augusta, GA 30912, USA; avakiti@augusta.edu (A.V.); vkota@augusta.edu (V.K.); gajebo@augusta.edu (G.H.A.); 6 Department of Medicine, Pathology, Medical College of Georgia, Augusta University, Augusta, GA 30912, USA; rkolhe@augusta.edu

**Keywords:** multiple myeloma, epidemiology, etiology, risk factors, incidence, mortality, diagnosis, staging, treatment

## Abstract

Multiple myeloma (MM) is a plasma cell disorder that is on the rise throughout the world, especially in the US, Australia, and Western Europe. In the US, MM accounts for almost 2% of cancer diagnoses and over 2% of cancer deaths (more than double the global proportion). Incidence has risen by 126% globally and over 40% in the US since 1990, while global mortality has risen by 94% and US mortality has fallen by 18%. The 5 year survival in the US has more than doubled over the past decades with the introduction of new targeted therapies and transplant techniques. Risk factors for MM include age (average age of diagnosis is 69), race (African Americans are over double as likely to be diagnosed), sex (men are at a 1.5× risk), and family history. Diagnosis includes serum or urine electrophoresis and free light-chain assay but requires bone marrow biopsy. It is distinguished from smoldering myeloma and monoclonal gammopathy of undetermined significance by a high (>3 g/dL) level of M-protein (monoclonal light chains) and the presence of CRAB (Hypercalcemia, Renal failure, Anemia, Bone pain) symptoms, which include hypercalcemia, renal failure, anemia, and bone pain, suggesting an end-organ damage. International staging system staging involves beta 2 microglobulin and albumin levels, while the revised system considers prognostic factors such as lactate dehydrogenase levels and chromosomal abnormalities. Front-line management includes induction regimen, maintenance therapy and hematopoietic cell transplantation for eligible patients and bisphosphonates or bone-stimulating agents for the prevention of skeletal events. Treatment for relapsed disease includes newly approved monoclonal antibodies like the CD38-targeting daratumumab, proteasome inhibitors, immunomodulating agents, and investigational therapies such as B cell maturation antigen Chimeric antigen receptor T cells.

## 1. Introduction

Multiple myeloma (MM) is a malignancy of plasma cells which is rising in incidence in the developed world. Plasma cells are mature antibody-producing B cells which reside in the bone marrow and are essential for maintaining humoral immunity. Multiple myeloma is characterized by a monoclonal proliferation of plasma cells resulting in the production of monoclonal antibody and end-organ damage [1]. This can damage bone marrow, resulting in cytopenia and frail, brittle bones, or renal failure [2]. The accumulation of the monoclonal antibody, Bence–Jones proteins, can precipitate in the urine resulting in kidney damage (usually type 2 renal tubular acidosis) and renal failure, and it can be seen in two-thirds of MM cases [2]. Multiple myeloma also activates osteoclasts in the bones through the nuclear factor kappa-B ligand (RANKL), resulting in the destruction of bone via lytic lesions that predispose to pain, fractures, and mobility issues, and calcinosis. In fact, the hallmark end-organ damage of MM is referred to as “CRAB” symptoms: hypercalcemia, renal involvement, anemia, and bone lesions [3].

Plasma cell dyscrasias are classified based on the occurrence of these symptoms and levels of monoclonal antibodies (most commonly IgG, but can also be IgM, IgA, or very rarely IgD). Those with the proliferation of plasma cells in their bone marrow and high antibody levels (M-protein), but without CRAB symptoms, are classified as smoldering myeloma (SM) [4]. Those without symptoms and with an M-protein below 1.5 g/dL are classified as plasma cell monoclonal gammopathy of undetermined significance (MGUS). Both SM and MGUS are considered precursors to MM with a 10% and 1% risk of progression per year, respectively [5]. Waldenstrom macroglobulinemia presents with high monoclonal IgM levels but is a rare proliferation of lymphoid cells (i.e., a B cell lymphoma as opposed to a plasma cell dyscrasia) and, thus, does not carry an increased risk for MM [6].

## 2. Epidemiology

### 2.1. Incidence

**Figure 1:**
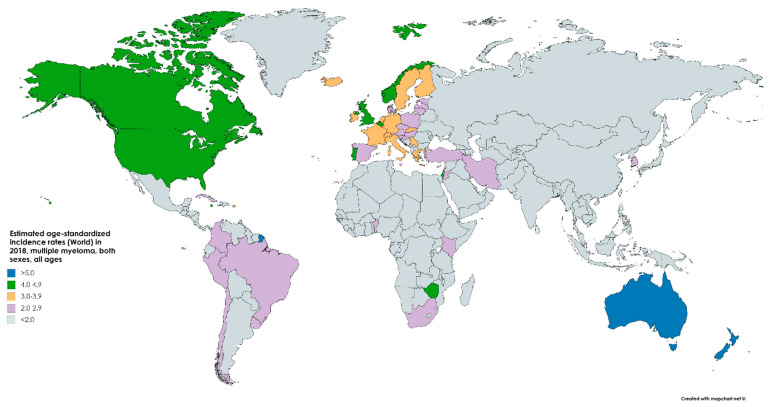
Map showing the estimated age-standardized incidence rates per 100,000 (world) in 2018 of multiple myeloma (MM) for both sexes and all ages. Created with mapchart.net. Data obtained from GLOBOCAN 2018 [7].

According to the latest Global Cancer Observatory (GLOBOCAN) statistics, there were an estimated 160,000 cases of MM globally in 2018, accounting for 0.9% of all cancer diagnoses (Figure 1) [7]. Approximately 90,000 of those cases were male and 70,000 were female, which equals an age-standardized incidence of 2.1/100,000 and 1.4/100,000, respectively. The cumulative risk of being diagnosed from birth to 74 is 0.24% among men and 0.17% among women, making the disease about 1.5× more likely in men [8]. From 1990 to 2016, the global incidence of MM increased by 126%. In 2016, MM cost 2.3 million disability-adjusted life years [9].

It is more common, and rising in incidence, in the developed world with the highest incidence in Australia, Western Europe, and the US. In the US, an estimated 32,000 cases were estimated to be diagnosed in 2020, accounting for 1.8% of all cancer diagnoses. This makes multiple myeloma the 14th most common neoplasm. The current estimated incidence rate, 7.0/100,000, is a 143% increase since 1975, when the incidence was 4.9/100,000 [10].

### 2.2. Mortality

**Figure 2:**
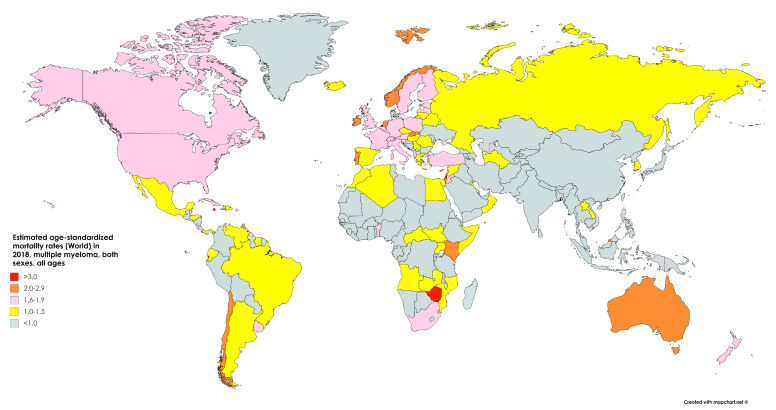
Map showing the estimated age-standardized mortality rates per 100,000 (world) in 2018 of multiple myeloma for both sexes and all ages. Created with mapchart.net. Data obtained from GLOBOCAN 2018 [7].

In 2018, an estimated 106,000 people globally perished of MM, accounting for 1.1% of all cancer deaths (Figure 2) [7]. Approximately, 59,000 of those deaths were male and 47,000 were female, equaling an age-standardized mortality of 1.3/100,000 and 0.9/100,000, respectively. The risk of death from MM was 0.15% among men and 0.10% among women, resembling the sex discrepancy in cancer risk (and suggesting similar survival between the sexes globally) [7]. Global deaths due to the neoplasm increased by 94% from 1990 to 2016 [9].

**Figure 3:**
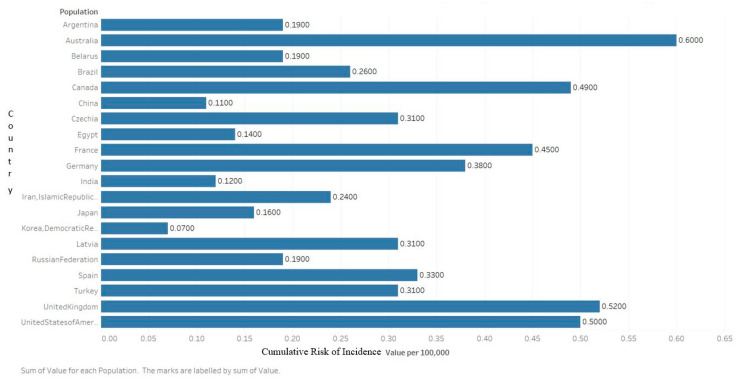
Graph showing the estimated cumulative risk of incidence in 2018 of multiple myeloma for both sexes, ages 0–74. Created with Tableau 2019.2. Data obtained from GLOBOCAN 2018 [7].

**Figure 4:**
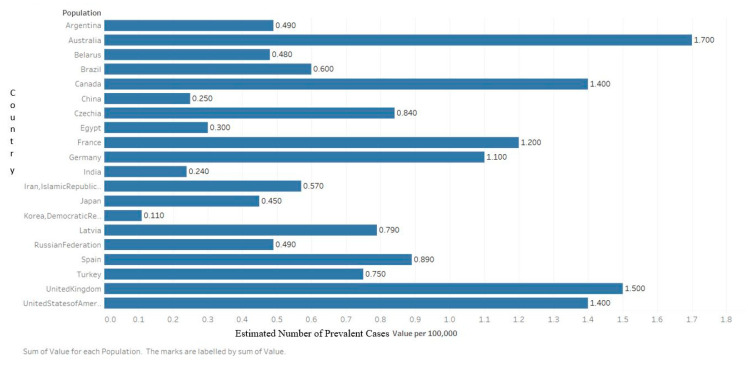
Graph showing the estimated number of prevalent cases (5 year) as a population in 2018 of multiple myeloma for both sexes, ages 0–74. Created with Tableau 2019.2 Data obtained from GLOBOCAN 2018 [7].

In the US, 12,800 people were estimated to perish in 2020 from MM, accounting for 2.1% of all cancer deaths. While incidence has risen over the past decades, mortality has fallen due to the drastically increased survival. The current mortality of 3.3/100,000 is 18% below the peak of 4.0/100,000 in 1994. This reflects a 2.27× increase in 5 year survival over the past decades, from 23.7% in 1976 to 53.9% in 2016 [10]. According to the Surveillance, Epidemiology, and End Results (SEER) program, the overall mortality rate dropped for all ages from 3.3/100,000 in 2013 to 3.2/100,000 in 2017, and from 21.7/100,000 in 2013 to 20.5/100,000 among greater than 65 age groups (Figure 3 and Figure 4) [11].

Survival is somewhat dependent on the stage at diagnosis, with a 74.8% 5 year survival for those with localized disease (which only accounts for 5% of all cases) and a 52.9% for systemic MM (the remaining 95% of diagnoses) [10]. Atkin et al. have shown that the prevalence of MGUS was much higher among emergency hospital admissions. They tested 660 patients, of which the overall rate of MGUS was 5.3%. The prevalence of MGUS in those aged > 50 years was 6.94%, higher than the previously published rate of 3.2%. There were higher rates in those with chronic kidney disease heart failure, anemia, or leukocytosis [12].

### 2.3. Risk Factors

#### 2.3.1. Age

**Figure 5:**
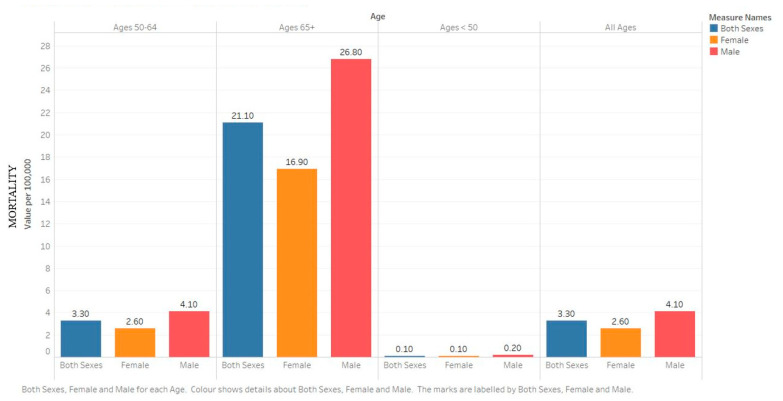
Bar chart showing the 5 year age-adjusted mortality rates from 2013–2017 by age for the United States. Created with Tableau 2019.2. Data source: SEER*Explorer [11].

Multiple myeloma is a neoplasm of older adults with the median age at diagnosis being 69 in the US. Over 60% of diagnoses are made in those greater than 65, and less than 15% of diagnoses are made in those under 55 (Figure 5) [11]. The median age of death is 75 with approximately 80% of deaths in those over 65 [10]. The accumulation of mutations that culminates in MM likely requires decades, making the disease only clinically visible among older adults, in the absence of other predisposing risk factors or mutations [1].

#### 2.3.2. Sex

It is about 1.5× more common among men than women, globally. Suggested underlying factors include discrepancies in health-risk behaviors, such as smoking and alcohol consumption, and higher rates of obesity among men, though none of these risk factors have been proven in MM [9].

#### 2.3.3. Race

**Figure 6:**
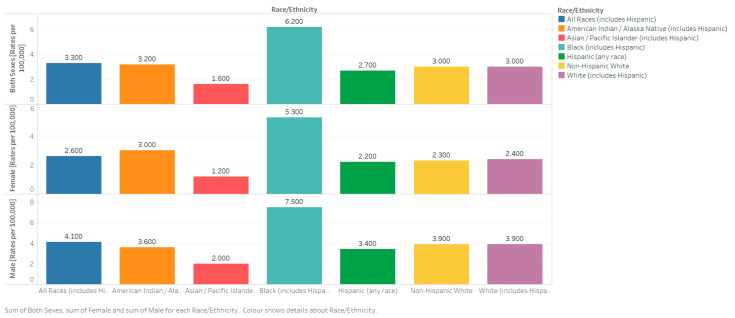
Bar chart showing the United States’ 5-year age-adjusted mortality rates from 2013–2017 for all races (including Hispanics). Created with Tableau 2019.2. Data source: SEER*Explorer [11].

It is more than twice as common among African Americans with an incidence of 16.5/100,000 among African American men and 12.0/100,000 among African American women (compared to 8.2 and 5.0, respectively, for Caucasians). This discrepancy is over 3× among those below 50, showing that African Americans have a younger onset of disease, on average [13]. Asians and Pacific Islanders were at a decreased risk, at 5.0 and 3.2 for men and women, respectively. African Americans were at a proportionately higher risk of death from MM with 7.5 and 5.3/100,000 deaths among African American men and women, respectively, as compared to 3.9 and 2.4 among Caucasians [10]. Retrospective studies from single institutions as well as the South West Oncology Group (SWOG) found no differences in survival based on race [14]. A study that used genetic profiling and fluorescent in situ hybridization (FISH) testing to search for the genetic foundations of the greater risk among African Americans did not find significant results [15]. Other studies have uncovered racial heterogeneity, such as a lower proportion of IgM MM or MGUS, among African Americans but have failed to explain the marked differences in disease risk [16]. The United States’ 5 year age-adjusted mortality rates by race, ethnicity, sex, and all ages from 2013–2017 are as shown in Figure 6 [11].

#### 2.3.4. Family History

While family clusters of MM have been described, underlying predisposing germline mutations have yet to be characterized. Retrospective results from the Multiple Myeloma Consortium found that the odds ratio (OR) for MM among first-degree relatives was 1.90, (95% CI: 1.26–2.87) with an especially strong association among men (OR = 4.13, 95% CI: 2.17–7.85) and African Americans (OR = 5.52, 95% CI: 1.87–16.27) [17].

## 3. Diagnosis, Staging, and Grading

### 3.1. Diagnosis

Patients often present with CRAB symptoms. When MM is suspected, blood and urine electrophoresis should be performed to look for the monoclonal light-chain secreted by the neoplasm. Blood levels of IgG, IgM, and IgA can identify the isoform of light-chain produced. If levels are elevated but under 3 g/dL, the disease can be classified as SM or MGUS instead of MM. A serum-free light-chain (FLC) assay is more sensitive than urine (where the light-chain protein is called a Bence–Jones protein). Serum albumin and beta-2 microglobulin (B2M) from bloodwork are also valuable for diagnosing and staging the disease. The definitive diagnosis requires a bone marrow biopsy with greater than 10% clonal plasma cells, or the presence of a plasmacytoma elsewhere. End-organ damage is necessary to distinguish from SM. Renal indices should be evaluated to a glomerular filtration rate, usually calculated by creatinine levels, that can be used to establish renal insufficiency. Imaging, such as CT (without contrast dye due to renal damage), MRI, and PET scans, is used to uncover lytic bone lesions. If patients are unable to undergo these imaging procedures, a skeletal survey is used instead. Eighty percent of patients have some skeletal lesions, fractures, or osteopenia at the time of diagnosis [3,18,19]. The revised diagnostic criteria by the International Myeloma Working Group Diagnostic Criteria for MM and related Plasma Cell disorders are shown in Table 1 [20].

### 3.2. Staging

The two main staging systems used for MM are the international staging system (ISS) and the Durie–Salmon system (DSS). The ISS stratifies cases into three stages: Stage I for those with a B2M less than 3.5 mg/L and albumin > 3.5 g/dL, Stage III for those with a B2M greater than 5.5 mg/L, and Stage II for all those in between [19]. The revised ISS adds prognostic information such as lactate dehydrogenase (LDH) levels and chromosomal abnormalities detected via FISH. In an international clinical trial with 3060 participants, for those with stage I disease, overall survival (OS) and progression-free survival (PFS) at five years was 82% and 55%, respectively. Median PFS was 66 months, and OS was not reached. For Stage II disease, these values were 62% and 36%, 42 months and 83 months, respectively. For Stage III disease, these values were 40%, 24%—29 months and 43 months, respectively. Further risk stratification can be made based on chromosomal translocation, many of which involve the IgH locus on chromosome 14 (14q32) [20].

With the advent of new therapies, the overall survival of the disease has increased. With improving survival, there was a need for re-staging the disease for early diagnosis, treatment, and preventing end-organ damage [20]. Until recently, MM was defined by the presence of a clonal process that correlates with end-organ involvement (presence of CRAB features). In patients with the absence of CRAB criteria, three biomarkers were included in the diagnostic criteria by the International Myeloma Working Group (IMWG) in 2014 [20]. In addition, the recommendations were to include CT and PET-CT to diagnose the involvement of bones. A new staging system has been developed that incorporates high-risk cytogenetic abnormalities in addition to standard laboratory markers of prognosis [20].

Both the ISS and DSS systems assess the tumor burden, but neither ISS nor DSS takes into consideration the biology of the disease, which determines the overall survival [20,21,22].

The revised international staging system (RISS) combines elements of tumor burden (ISS) and disease biology [23]. It was developed based on a study of 11 international trials. The 5 year survival rates among the patients with Stage I, II, and III RISS were 82%, 62%, and 40%, respectively [20].

### 3.3. Management

**Figure 7:**
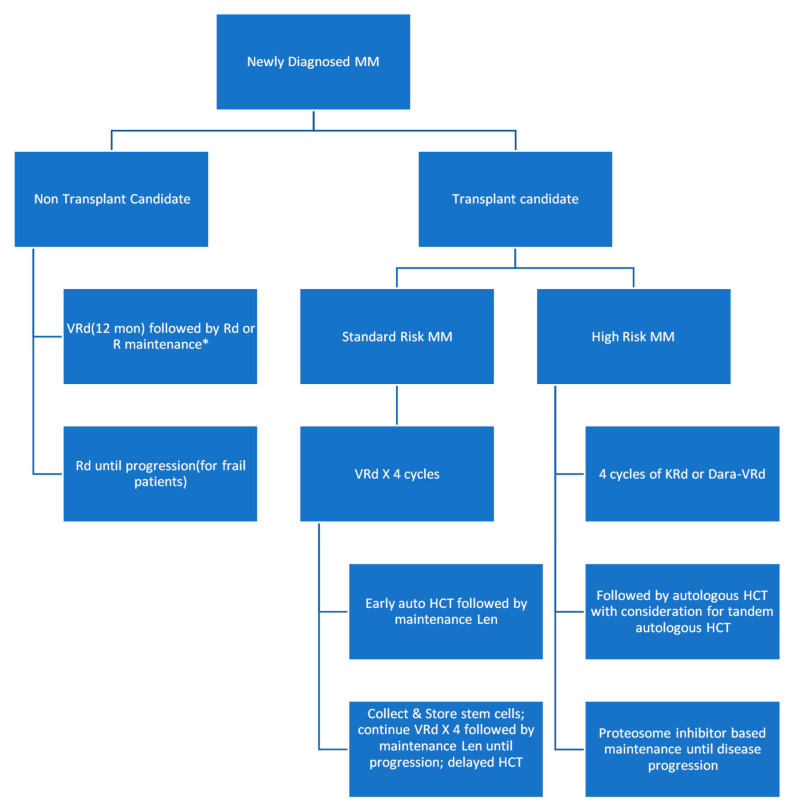
Modified flow chart depicting the general outline of treatment options for MM adopted from mSMART.org. HCT: hematopoietic stem cell transplantation.

The survival has more than doubled over the past decades due to the introduction of new chemotherapy combinations, targeted small molecule inhibitors, and monoclonal antibodies. In Figure 7, we present a modified flow chart depicting the general outline of treatment options for MM adopted from mSMART.org. The approval of multiple active agents in the treatment of MM has generated numerous possible drug combinations that can be used in first-line and relapsed settings. The initial therapy of patients with symptomatic MM depends on risk stratification of the MM, the patient’s eligibility for autologous hematopoietic stem cell transplantation (HCT), and an assessment of the patient’s pre-existing comorbidities (e.g., presence of neuropathy or renal failure). For those ineligibles for transplant, a triplet regimen is also recommended and then followed with maintenance therapy until toxicity limits the use. One of the most commonly used front-line triple therapies is bortezomib (a proteasome inhibitor), lenalidomide (an immunomodulator that downregulates inflammatory and proliferative cytokines), and dexamethasone (a long-acting steroid), (called VRd after the tradenames Velcade, Revlimid, and Dexamethasone, respectively) [18,24].

The results of the SWOG S0777 trial showed that the triplet regimen VRd (i.e., bortezomib, lenalidomide, dexamethasone) improved response rates, depth of response, progression-free survival, and overall survival compared with the then approved front-line regimen Rd (lenalidomide, dexamethasone) making a three-drug regimen the main stay of initial therapy for most patients with MM [25]. This induction is typically followed by lenalidomide and dexamethasone maintenance. As of 2016, lenalidomide and bortezomib have been approved in 73 and 106 countries worldwide, respectively.

For most patients with standard-risk MM, the most commonly used induction therapy is VRd. VCd also called CyBorD (bortezomib, cyclophosphamide, dexamethasone) is an acceptable alternative for patients who are taught to have an increased risk of complication from the use of lenalidomide (e.g., patients with acute renal failure or those with increased thromboembolic risk) [26]. Other triplets like DRd (daratumumab, lenalidomide, and dexamethasone) and KRd (carfilzomib, lenalidomide, and dexamethasone) are also reasonable options for patients who cannot tolerate bortezomib (e.g., due to the fact of severe neuropathy) and for those who are not transplant candidates. Although OS data are immature, DRd has shown improved PFS when compared with Rd [27]. For frail patients who may not tolerate the increased toxicity that comes with triple regimens, Rd is still an acceptable alternative.

If patients relapse within 12 months of completion of initial therapy, this is a considered “high-risk” relapsed disease. Patients with a high risk of MM do poorly with conventional treatment options and are therefore encouraged to participate in clinical trials that employ novel therapeutic strategies. Outside of a clinical trial, VRd remains the preferred choice of initial chemotherapy for patients with high-risk MM who are not candidates for HCT [28,29]. KRd instead of VRd can be used as initial therapy in patients with t(4;14), t(14;16), t(14;20), and those with ≥2 high-risk abnormalities based on the results of phase 2 studies suggesting higher complete response (CR) rates and minimal residual disease negativity rates compared with historical results using VRd [28,30,31]. Albeit weak, there is data suggesting that use of a proteasome inhibitor may abrogate the prognostic impact of at least some of these high-risk genetic markers [32].

On the front line, autologous HCT is preferred for those who can tolerate it. Those undergoing HCT typically receive a triplet regimen for 3–4 months prior to stem cell collection in order to reduce tumor cell numbers in the bone marrow. Those who had a durable benefit from HCT in front-line or did not receive HCT in front-line but are now eligible are recommended an HCT in second line following induction [33,34]. Collected cells can be transplanted early or on a delayed schedule (after 8–12 cycles of the initial therapy or after first relapse). Allogeneic transplant remains under investigation for MM.

The recently approved monoclonal antibody, daratumumab, which targets CD38 (a receptor highly expressed on myeloma cells), has become a favorite for later line induction regimens [35,36]. Other antibodies approved include elotuzumab for the SLAMF7 receptor and isatuximab, which also targets CD38 (but comes with a greater risk of adverse events since it is chimeric). In addition, also used in relapsed myeloma are next generation proteasome inhibitors such as carfilzomib and ixazomib. Multiple myeloma relies on proteasomes to eliminate protein waste caused by ceaseless monoclonal light-chain production, thus inhibiting proteasomes selectively targets myeloma cells. Lastly, immunomodulators like lenalidomide, pomalidomide, and thalidomide are frequently used in triplet or quadruplet induction regimens and maintenance regimens [33,34].

Extramedullary disease can be seen in 5% of MM patients [37,38]. It can manifest in the skin, lymph nodes, abdomen, upper respiratory tract, and the central nervous system (CNS) [39].

Despite significant advances in the management of myeloma, the treatment for relapsed/refractory multiple myeloma (RRMM) continues to be challenging. Venetoclax, an inhibitor of the anti-apoptotic protein BCL-2, is very effective among patients with t(11;14). A review by Basali et al. [40] has shown an overall response of approximately 78% during their experience with venetoclax-based regimens in ten RRMM patients who had a median of six prior lines of therapy. Central nervous system involvement is not only a rare complication but also has a very poor prognosis. Recently, Egan et al. [41] provided a review of the current literature including the presentation, treatment, and survival data among patients with CNS involvement.

Other treatment considerations, including bisphosphonates, such as zoledronic acid, or bone-stimulating agents (BSAs), like denosumab, are recommended upon diagnosis in order to prevent or palliate lytic lesions and fractures [42,43]. Moreover, there is the need to use antiviral prophylaxis with acyclovir 400 mg BID throughout treatment with proteasome inhibitors, administration of bortezomib subcutaneously rather than IV, and once weekly rather than twice weekly to dramatically decrease the risk of peripheral neuropathy without compromising efficacy [44,45,46,47]. When using regimens that include lenalidomide (such as VRd, Rd, and KRd), consideration for the need of routine antithrombotic prophylaxis, dose adjustment for patients with creatinine clearance < 60, and recognition of the myelotoxic nature of lenalidomide to the bone marrow making peripheral stem cell collection difficult is very important. Low-dose dexamethasone (i.e., once weekly 40 mg dose) is associated with less dexamethasone-related toxicity than more intensive dosing scheme without compromising efficacy [48].

Several studies are underway to evaluate the effect of adding a fourth drug (such as daratumumab) to a triplet backbone but are not yet standard of care. Chimeric antigen receptor T cell therapy for the MM receptor BCMA is currently being investigated and has shown promise for refractory disease [49].

## 4. Complications

With the advancement in the treatment options, myeloma is more like a chronic disease and the goal of the therapy is to prevent end-organ damage and to attain disease-free survival [50]. The complications could be secondary to the disease itself or drug induced. Kidney involvement is very common in myeloma and other plasma cell dyscrasias. Plasmapheresis is considered in patients with cast nephropathy to reduce the concentration of the paraproteins, but its efficacy has not been well established. It does not have any effect on overall survival or need for hemodialysis; however, the patients with hyperviscosity syndrome do benefit with plasmapheresis [51]. Up to 40% of the patients showed renal recovery when treated with proteasome inhibitors and immunomodulators and has shown to have a superior OS when compared to the non-responders [52]. Multiple myeloma is one of the common causes of hypercalcemia secondary to malignancy [53].

The patients are at a higher risk of infection due to the disease and immunosuppression secondary to chemotherapy. Neurological complications include peripheral neuropathy and cognitive issues secondary to therapy. Cognitive deficits can be noted in patients treated with immunomodulators [54,55]. There is an increased risk for the development of both secondary hematologic and solid organ cancers, especially in post-transplant maintenance with lenalidomide [56]. There is also an increased incidence of venous thromboembolism compared to the general population [57]. Cardiac complications, such as heart failure, chest pain, arrhythmias, and pulmonary hypertension, can be seen in patients when treated with carfilzomib [58,59].

## 5. Conclusions

Multiple myeloma is an aggressive plasma cell dyscrasia that is on the rise in the US and much of the developed world. In the US, MM constitutes over 2% of all cancer deaths (which is double the proportion in the rest of the world). While incidence has grown by over 40% in the US over the past decades, mortality has fallen, and 5 year survival has more than doubled thanks to the introduction of new therapies and transplant techniques. The neoplasm is more common among older adults, men, and African Americans, and has been shown to have a hereditary component to risk. The first step in diagnosis is urine or serum electrophoresis and FLC assays, followed by imaging. Multiple myeloma is distinguished from SM and MGUS by a high (>3 g/dL) level of M-protein (monoclonal light chains) and the presence of CRAB symptoms (end-organ damage). The ISS staging was originally based on B2M and albumin levels, while the revised system includes prognostic factors such as LDH levels and chromosomal abnormalities. Front-line management includes HCT for those who are eligible, induction triplet regimens, and maintenance therapy, along with bisphosphonates or BSAs for prevention of skeletal events. Treatment for the relapsed disease includes monoclonal antibodies like the CD38-targeting daratumumab, proteasome inhibitors, and immunomodulating agents.

Objectives and Novelty: This article describes the latest review of multiple myeloma. An extensive literature search was done and up-to-date information regarding epidemiology, staging, and management was described.

## Figures and Tables

**Table 1:** The revised diagnostic criteria by the International Myeloma Working Group Diagnostic Criteria for MM and related Plasma Cell disorders [20]. CRAB: Hypercalcemia, Renal failure, Anemia, Bone pain; FLC: free light chains; MGUS: monoclonal gammopathy of undetermined significance.

Disorder	Diagnostic Criteria
SMOLDERING MULTIPLE MYELOMA	Two criteria must be met: (1) Serum monoclonal protein (IgG or IgA) 3 gm/dL or more, or urinary monoclonal protein 500 mg or more per 24 h and/or clonal bone marrow plasma cells 10–60% (2) No evidence of myeloma-defining events or amyloidosis
MULTIPLE MYELOMA	Two criteria must be met: (1) Clonal bone marrow plasma cells more than 10% or biopsy-proven bony or extramedullary plasmacytoma Plus, one or more of the following myeloma-defining events: (1) Presence of CRAB criteria which can be attributed to the underlying plasma cell proliferative disorder, specifically: - Hypercalcemia: serum calcium >11 mg/dL - Renal insufficiency: creatinine clearance <40 mL/min or serum creatinine >2 mg/dL - Anemia: hemoglobin <10 g/dL or >2 g/dL below the lower limit of normal - Bone lesions: one or more osteolytic lesions on skeletal radiography, CT, or PET-CT (2) Clonal bone marrow plasma cell percentage 60% or more (3) Involved/uninvolved serum FLC ratio of 100 or more (involved FLC level must be 100 mg/L or more) (4) More than one focal lesion on MRI studies (at least 5 mm in size)
IgM MGUS	Three criteria must be met: (1) Serum IgM monoclonal protein less than 3 gm/dL (2) Less than 10% bone marrow lymphoplasmacytic infiltration (3) No evidence of anemia, constitutional symptoms, hyperviscosity, lymphadenopathy, or hepatosplenomegaly secondary to underlying lymphoproliferative disorder
LIGHT-CHAIN MGUS	All the following criteria must be met: (1) Abnormal FLC ratio (Less than 0.26 or more than 1.65) (2) Increased level of the appropriate involved light chain (increased kappa FLC when the ratio is more than 1.65, and lambda FLC in patients if the ratio is less than 0.26) (3) Absence of immunoglobulin heavy-chain expression on immunofixation (4) No evidence of end-organ damage that can be attributed to the plasma cell proliferative disorder (5) Clonal bone marrow plasma cells less than 10% (6) Urinary monoclonal protein less than 500 mg/24 h

## Data Availability

The authors declare that data supporting the findings of this study are available within the article.

## Abbreviations

MM	Multiple myeloma
SM	smoldering myeloma
MGUS	monoclonal gammopathy of undetermined significance
CRAB	hypercalcemia, renal failure, anemia, bone involvement
ISS	international staging system
B2M	beta 2 microglobulin
LDH	lactate dehydrogenase
HCT	hematopoietic cell transplantation
BSAs	bone-stimulating agents
GLOBOCAN	Global Cancer Observatory
SEER	Surveillance, Epidemiology, and End Results
FLCs	Free light chains
VRd	bortezomib, lenalidomide, dexamethasone
Rd	lenalidomide, dexamethasone
VCd also called CyBorD	bortezomib, cyclophosphamide, dexamethasone
DRd	daratumumab, lenalidomide, and dexamethasone
KRd	carfilzomib, lenalidomide, and dexamethasone
RRMM	relapsed/refractory multiple myeloma
CNS	central nervous system
IMWG	International Myeloma Working Group
OS	overall survival
PFS	progression-free survival
DSS	Durie–Salmon system
BCMA-CART	B cell maturation antigen Chimeric antigen receptor T cells
